# Rethinking Geoengineering Governance Utilizing the Playing God Argument: Considerations of Knowledge, Control, and Benevolence

**DOI:** 10.1007/s11948-025-00569-6

**Published:** 2025-12-01

**Authors:** Julian Dreiman, Brian Patrick Green

**Affiliations:** 1 Independent Scholar, San Francisco, California USA; 2 Markkula Center for Applied Ethics, Santa Clara University, 500 El Camino Real, Santa Clara, California USA

**Keywords:** Governance, Ethics, Geoengineering, Climate change

## Abstract

Because greenhouse gas emissions continue to grow, it is increasingly challenging to limit global warming to 1.5 or 2.0 degrees Celsius. As such, there is a growing debate on whether or not to deploy geoengineering to reduce warming. Geoengineering is controversial, and many arguments have been raised against it, including the “playing God” critique. When understood through the philosopher Moti Mizrahi’s reinterpretation, the playing God critique does not eliminate the possibility of using geoengineering, but rather informs how its governance can be improved. We use Mizrahi’s interpretation of the playing God critique to claim that the existing governance literature lacks sufficiently actionable proposals which duly incorporate three components of effective governance: control, knowledge, and benevolence. We then go on to suggest some actionable governance mechanisms aimed at supporting the benevolent use of geoengineering. We believe that a three legged approach to geoengineering governance can both respond to the playing God critique and encourage the development of a more robust, well rounded governance regime.

## Introduction

It is becoming increasingly challenging to limit global warming to 1.5 or 2.0 degrees Celsius. The debate on whether or not to deploy geoengineering to actively reduce warming is, therefore, becoming increasingly important. Many arguments have been raised against geoengineering, including the “playing God” critique, which rejects certain kinds of technological interventions as inappropriate for humanity because they are “playing God” – i.e., using them is not something humans should do. These complaints could be dismissed as superstition, but this would be a disservice to those concerned with this line of response, and would deny the world a chance to examine the “playing God” core concern, which, as we shall see, is not actually theological in nature. Summary rejection of popular concerns merely aggravates the multitudes of people who understand the argument intuitively, but have not yet reflected upon their fears enough to raise them to the level of carefully elucidated discourse. This paper hopes to do some of this work, examining the rational core of the “playing God” argument, and then determining what relevance it has, if any, for geoengineering governance.

In his investigation of this particular criticism of humans’ use of technology, philosopher Moti Mizrahi identifies a key component of the argument: humans are neither omnipotent, nor omniscient, nor omnibenevolent (Mizrahi, 2020). When applied to geoengineering, Mizrahi’s interpretation of the playing God critique does not entirely dismiss its use, but rather highlights how its governance might be improved by increasing humans’ control of, knowledge about, and ability to benevolently use it.

We use Mizrahi’s interpretation of the playing God critique because it offers a constructive lens through which to view geoengineering without primarily arguing that it does or doesn’t amount to playing God. Building from Mizrahi, we claim that to be more effective, geoengineering governance should have three components: an element aimed at improving humans’ control over the technology, another element aimed at increasing their knowledge about it, and lastly an element aimed at expanding human capability to benevolently use and adapt to geoengineering. We identify that the current governance literature has a relative lack of actionable mechanisms for directing geoengineering benevolently (at least compared to those focused on control and knowledge), thus we propose specific mechanisms aimed at closing that gap. We believe that this three-legged approach to governance can both respond to the playing God critique and encourage the development of a more robust, well rounded governance regime.

In section one, we begin by defining geoengineering and categorizing different types of climate interventions. Then, in section two, we explore different interpretations and uses of the playing God critique. In section three, we focus on a more recent interpretation of the playing God critique and explore its implications through a case study of stratospheric aerosol injection. In section four, we highlight existing governance proposals and note gaps. Finally, in section five, we suggest four proposals which address the Playing God critique and advance the geoengineering governance debate.

## Geoengineering

The Royal Society defines geoengineering as “the deliberate large-scale manipulation of the planetary environment to counteract anthropogenic climate change” (The Royal Society, 2009). While geoengineering-related research has been conducted to some degree for many decades, scholars note that Paul (Crutzen’s, 2006 essay, “Albedo enhancement by stratospheric sulfur injections: A contribution to resolve a policy dilemma?” broke the taboo on research and ushered in the modern era of investigation (Crutzen, 2006).

Broadly understood, there are two types of geoengineering: carbon dioxide removal (CDR) and solar radiation management (SRM) (Hamilton, 2013). While there are overlapping governance challenges and approaches shared by CDR & SRM, this paper focuses exclusively on SRM as it’s understood to be cheaper and quicker than CDR, has greater variance in possible outcome, and because much of the governance literature already focuses on SRM. For instance, a 2020 literature review by Pamplany et al. found that of 304 geoengineering ethics papers, 86 focused exclusively on SRM whereas only 21 were exclusive to CDR (Pamplany et al., 2020).

Solar radiation management seeks to reduce the amount of the sun’s energy entering the atmosphere thereby limiting the greenhouse effect and lowering global surface temperatures (Rasch et al., 2008). Several approaches to SRM have been discussed such as marine cloud brightening, cirrus cloud thinning, and space-based reflectors. The most commonly researched — and most likely to be deployed — is stratospheric aerosol injection (SAI) (Parson & Keith, 2024). Stratospheric aerosol injection interventions propose distributing sulfate aerosols into the stratosphere 10–50 KM above earth’s surface with the intention of reflecting incoming solar radiation (Hamilton, 2013).

SAI research is primarily based on using earth system models to predict the effect of adding sulfur, or other elements, into the stratosphere. Specific to SRM research, scholars use the Geoengineering Model Intercomparison Project to standardize their model experimentation (Kravitz et al., 2011). Modeling is the primary research avenue because outdoor testing is both technically challenging and controversial. For example, both Harvard’s SCoPEx and the UK’s SPICE projects which aimed for real world testing were shut down before they could begin (Temple, 2024; Bodle et al., 2014). It’s worthwhile to note that the distinction between geoengineering research and deployment is hazy. Alan Robock et al. argue, for example, that SAI cannot be accurately studied without a full scale implementation (Robock et al., 2010).

A more thorough assessment of the concerns, uncertainties, and harms associated with SAI is presented in Section “Mizrahi’s playing god and stratospheric aerosol injection (SAI)”, but we offer an introductory overview here. It is important to note that while SAI can create climate conditions similar to low emissions scenarios, such as lowering surface temperatures, it can never fully reverse the effects of greenhouse gas emissions (Irvine et al., 2016). Furthermore, it is crucial to understand that SAI’s degree of effect is challenging to predict. Predictions can only be estimates.

One predicted effect of SAI is that it will impact precipitation. For example, in their 2016 review of solar geoengineering’s effect on earth system science, Irvine et al. note that under SAI, high intensity rainfall events would be less common and low intensity rainfall more common. Further, they find that overall rainfall would likely decrease, but that evaporation would decrease more, resulting in a net run off increase. In short, SAI will impact many earth and climate systems including: atmospheric heating, stratospheric chemistry, the quality of light, crop production, storm pattern and strength, and ocean acidity — to name just a few (Ricke et al., 2023)

While it’s highly likely that SAI will impact earth systems, the causal mechanism of that impact is less certain (Hulme, 2012). One complication worth mentioning is that there is no consensus on which kind of sulfur particles are best suited, how many particles need to be injected into the stratosphere, at which altitude and latitude they ought to be applied, and for how long. For example, Philip Rasch et al. propose a million annual flights to deliver the needed amount of sulfur for SAI whereas Wake Smith and Gernot Wagner propose a gradual increase in the number of flights starting with just 4,000 (Rasch et al., 2008; Smith & Wagner, 2018).

At a higher level, Susanna Baur et al. show how SRM interventions – of which SAI is one – are impacted by “different emissions scenarios, net-negative emissions, and climate uncertainty” (Baur et al., 2023). Uncertainty, unpredictability, and disagreement are throughout the literature, yet even if all the literature were in agreement the field would still face accusations of “playing God.”

## Playing God

Clive Hamilton defines playing God as “humans crossing a boundary to a domain of control of causation that is beyond their rightful place” (Hamilton, 2013). While used broadly, the playing God critique is commonly leveled against new technologies when they appear to give humans an ability previously thought to only be held by God. It has been used to critique nanotechnology, cloning and genetic engineering, and as we explore below, geoengineering (Simons, 2022). But what does the playing God critique actually mean? One interpretation, formalized by Laura Hartman is that “(1) certain tasks are reserved for God, not humans; therefore, (2) if humans undertake these tasks they are doing wrong in some way, that is, they are ‘playing’ God without actually being God; and (3) bad consequences will likely result” (Hartman, 2017).

Playing God, however, can be understood through many different arguments and angles. Georgiana Kirkman, for instance, argues that playing God is perhaps better understood not through a deontological lens (i.e., humans should not play god) but rather through the virtue ethics lens (Kirkham, 2006). The critique, understood this way, implies that those who seek to play God are not acting in a virtuous manner. Adam Waytz and Liane Young posit a different basis of the critique, that people have an aversion playing God because doing so violates “the natural order of things” (Waytz & Young, 2019). The natural world has a certain sanctity which ought not be altered. Dale Jamieson offers yet another view which posits that modifying nature to conform to humanity’s needs and desires errs into excessive planetary control (Jamieson, 2013). Moreover, this excessive control shows human arrogance and their failure to respect nature’s order.

An additional interpretation of the playing God critique is proposed by Moti Mizrahi. Under Mizrahi’s interpretation, the aversion to playing God should be understood as less of a warning to “leave God’s creation or nature alone, but rather as an invitation to think carefully about all the ways in which the use of new technologies could go seriously wrong” (Mizrahi, 2020). This approach to understanding and applying the playing God critique is compelling because rather than blanketly arguing a technology should be off limits because it is under God’s domain – it provides a constructive lens through which to determine if a technology is suitable for human use. Additionally, it doesn’t presuppose the existence of God, but only the *idea* of God, thereby addressing the issue not from a potentially limited religious perspective, but from a philosophical perspective with strong cultural and psychological relevance. Finally, Mizrahi’s interpretation gives specificity to the playing God critique. Other formulations can be vague, whereas Mizrahi’s is tractable: one can score a technology against it.

Mizrahi proposes an argumentation scheme that invites people to determine if and how the use of a new technology could go wrong. First, however, he notes that traditional conceptions portray God as being omnipotent (all-powerful), omniscient (all-knowing), and omnibenevolent (all-good). Thus, if God were in charge of a new technology, God would never lose control of it, could foresee all consequences of using it, and have the moral compass to never misuse or abuse it. Humans, he notes, are not omnipotent, omniscient, or omnibenevolent and therefore to play God would be to pretend to be something that we are not.

Mizrahi’s scheme is as follows.

Premise 1: We should not (morally speaking) “play God.”

Premise 2: Using new technology *T* amounts to “playing God.”

Conclusion: We should not (morally speaking) use *T*.

Mizrahi’s argument rests on premise 2. Thus, he proposes three critical questions to determine if using a new technology amounts to playing God. The critical questions are as follows.

Critical Question 1: Could *T* get out of the control of (less than omnipotent) human beings?

Critical Question 2: Could *T* have unforeseen consequences for (less than omniscient) human beings?

Critical Question 3: Could *T* be misused or abused by (less than omnibenevolent) human beings?

Affirmative answers to one, two, or all of these questions *could* signal that the use of a new technology *does* amount to playing God, or at least that the analogy is suitable. Simultaneously, Mizrahi’s questions are not meant to be immediately definitive, rather they are meant to provoke an investigation of how humans control, create knowledge about, and benevolently use a certain technology. While Mizrahi’s view of playing God is illuminating, it does, however, have some practical limitations. First, the critical questions are broad and could be answered affirmatively for many, if not all, technologies. Taken to an extreme, one could use Mirazhi’s argumentation scheme to argue that use of any technology amounts to playing God. Second, premise 1, “we should not (morally speaking) ‘play God,’” may be colloquially understood, but it does not rest on an air-tight logical foundation. Mizrahi claims that it’s wrong to pretend to be something that you’re not (i.e., God), but perhaps a strong argument exists that humans should in fact seek to play God (i.e., pretend to be something they’re not).

Understanding these drawbacks, we still find utility in applying Mizrahi’s scheme to geoengineering to think carefully about how its use could go wrong, and highlight more effective and novel governance mechanisms. Mizrahi’s critical questions highlight specific aspects of governance that need to be addressed in a way that other interpretations of the playing God critique do not. Before addressing governance strategies, however, in the following section we apply Mizrahi’s argumentation scheme and critical questions to stratospheric aerosol injection.

## Mizrahi’s Playing God and Stratospheric Aerosol Injection (SAI)

Recognizing the diversity of SAI proposals, we will apply Mizrahi’s critical questions specifically to a proposal made by Smith and Wagner. Smith and Wagner propose to loft sulfur (which would then be combusted into sulfur dioxide gas) into the stratosphere at an elevation of 20 km and within the latitudes of 15 and 30 degrees North and South of the equator. They calculate that over a 15 year period, if roughly one tenth of a megaton (100,000 metric tons) of sulfur is released in the first year of SAI, and that the rate increases linearly, that the rate of surface temperature warming would reduce by half.

Thus, with this proposed SAI intervention in mind, we apply Mizrahi’s first critical question. *Could Smith and Wagner’s proposed SAI intervention get out of the control of (less than omnipotent) human beings?* We see compelling evidence that it could. It’s reasonable to assume that humanity might lose control of SAI by either releasing too much sulfur or too little sulfur, thereby rendering the SAI intervention useless or overly effective. While releasing too little sulfur could be solved by simply releasing more, interfering with the climate on a global scale is intrinsically difficult and has various known and unknown secondary and tertiary effects.

Scholars have noted that humanity could also lose control of SAI through a myriad of catastrophic events such as war, pandemic, or terrorism. If SAI were to suddenly and permanently stop, the world might experience “termination shock,” which is rapid warming of the planet caused by the accumulated greenhouse gas emissions that had been masked by SAI (Parker & Irvine, 2018). Termination shock is particularly concerning because humans, animals, and other natural systems would have less time to adapt to the quickened pace of warming. Andy Parker & Peter Irvine note, however, that SAI, which seeks to cool the planet by a few tenths of a degree Celsius, could be stopped suddenly without triggering termination shock. Controversially, they argue that maintaining SAI is technically easy and that once started there’s a global motivation to continue it. That said, many scientists and policy makers believe even the slight possibility of termination shock shows that humans cannot control SAI in a way that would satisfy the playing God critique.

A related concern about humans losing control of SAI is double catastrophe. Introduced in the context of geoengineering by Seth Baum et al., double catastrophe defines a scenario where a “catastrophic societal collapse eliminates society’s ability to continue SAI” thereby leading to termination shock (Baum et al., 2020). Needless to say, double catastrophe is more dangerous than just termination shock.

Turning to Mizrahi’s second critical question, we ask: *could Smith and Wagner’s proposed SAI intervention have unforeseen consequences for (less than omniscient) human beings?* Again, we find ample evidence that there could be many unforeseen consequences of the use of geoengineering by less than omniscient humans. Because the climate is a complex and ever changing system, it is difficult to fully determine how different factors affect the weather, ecosystems, and other animals.

Although not fully understood, scholars have identified certain systems whose normal functioning would be altered by SAI. One commonly studied system is the stratospheric ozone layer. Simone Tilmes et al. show that under certain earth system models, SAI would decrease the amount of ozone in certain high Southern latitudes while increasing it in mid and high Northern hemisphere latitudes (Tilmes et al., 2022). That said, they also conclude that the earth system models “show shortcomings in representing different processes properly” and call for improvements to earth system models to more accurately model SAI’s impacts. Another often discussed potential impact of SAI are changes to precipitation and weather patterns. One study about Africa’s river basins finds that SAI could create a climate water balance deficit – and possibly drought – in tropical regions (Abiodun et al., 2021).

Yet another area of impact is SAI’s effect on ecosystems, plants, and animals. In a comprehensive review, Phoebe Zarnetske et al. list many of the potential effects SAI could have on ecosystems (Zarnetske et al., 2021). For example, they note that cooling caused by SAI might “reduce nutrient cycling and ecosystem primary production” and that a possible increase in surface UV might cause “a decline in plant productivity.” Furthermore, they argue that SAI-induced cooling could alter vegetation distribution and wouldn’t decrease ocean acidification. Perhaps most illuminating, of the fact that scientists don’t fully understand SAI’s impact on ecosystems, is the authors’ conclusion that,

despite the development of SRM schemes and SAI scenarios for modifying Earth’s climate, little is known about how these scenarios would impact the health, composition, function, and critical services of ecological systems.

Lastly, we note that while there are many known unknowns regarding the impacts of SAI, additionally, it is highly likely that there are also “unknown unknown” impacts of SAI which would not be discovered until a full scale implementation began.

Considering Mizrahi’s final critical question, we ask: *could Smith and Wagner’s proposed SAI intervention be misused or abused by (less than omnibenevolent) human beings?* Again, we find evidence that the answer is yes. One clear way that SAI could be misused or abused is defined as the free driver problem. The free driver problem - not to be confused with the “free rider problem” - describes how one actor can unilaterally decide to deploy SAI, therefore imposing dangers or harms onto others (Heyen & Lehtomaa, 2021). Atop that concern is the worry of SAI over-provision. Because SAI is cheap, and deployment not necessarily coordinated, multiple individual actors may decide to use it causing above optimal levels of cooling (Moreno-Cruz et al., 2025). Related to the free driver problem is the concern that SAI could become militarized and used as a lever of state power for international competition (Hamilton, 2013). Hamilton notes that the US has long studied the military applications of “controlling the weather.”

Other ways that SAI could be misused or abused are described as the slippery slope and moral hazard concerns (Hamilton, 2013; Andow, 2023; Tang, 2023). The slippery slope concern is that once outdoor SAI research begins it will create inertia towards full scale deployment that would supersede moral or other objections against it. Related, opponents of SAI fear that deployment decisions would be made undemocratically therefore unfairly “locking in” the whole world to a geoengineered future. The moral hazard concern is that the possibility of deploying SAI – and therefore cooling the earth’s temperature – would dissuade people and governments from reducing greenhouse gas emissions in the first place and therefore increasing their future desire to deploy SAI (McLaren, 2016; Markusson et al., 2018). SAI opponents are wary of the fossil fuel industry’s influence in and support of geoengineering research.

Recognizing that the likely answers to Mizrahi’s critical questions are yes for SAI, we can presume the second premise in his argumentation scheme is true, and therefore conclude that there is a strong case against using SAI until we can ensure that humans are better able to control it, understand its effects, and use it benevolently. That said, these answers are not definitive, and perhaps a more thorough investigation of them (as Mizrahi intended) could yield different results. This call to investigatory action is especially compelling considering that geoengineering research and outdoor testing is increasing, and that societies’ understanding and views on the topic are evolving quickly (Flavelle, 2024). Mizrahi’s interpretation of the playing God critique – and the fact that research and outdoor testing are ongoing – show us that instead of blanketly arguing we ought not to use geoengineering technology, a more constructive approach would be to design governance that seeks to increase humans capacity to control, understand, and benevolently use it.

## Geoengineering Governance

The Geoengineering governance literature is broad, loosely defined, and quickly growing. We present a non-exhaustive summary of recent scholarship, and lean on Reynolds to define governance as the “goal-oriented, sustained and explicit use of authority to influence behavior” (Reynolds, 2019). Crucially, we note that few, if any, proposals thoroughly address the three necessary elements of governance as noted by Mizrahi: effective control, knowledge creation, and benevolent usage.

At the highest level, governments, NGOs, and international organizations have put forward high-level governance proposals. For example, in 2023, the Climate Overshoot Commission released the Reducing the Risks of Climate Overshoot report which suggests a moratorium on SRM deployment and large-scale outdoors experiments (Climate Overshoot Commission, 2023). Further, the commission calls for SRM research to be transparent and to *not be led* by for-profit institutions. Also in 2023, the Office of Science and Technology Policy shared a SRM research plan and governance framework that proposes four governance points for the US government to follow (OSTP, 2023). These include modeling responsible behavior, encouraging other entities to practice transparent research, asking federal agencies to adopt F.A.I.R.E.R. (Findable, Accessible, Interoperable, Reproducible, Equitable, and Responsible) data handling standards, and ensuring public consultation and periodic review.

In 2021, The National Academies released their Reflecting Sunlight report which offers a thorough assessment of, and proposal for, SAI research governance (National Academies of Sciences, Engineering, and Medicine, 2021). Its authors suggest that research governance ought to include compliance with laws, adherence to ethical norms, promotion of trust, and sharing of knowledge. They also propose specific recommendations such as a code of conduct, registry and data sharing, assessment and review, and permitting. In 2024, The American Geophysical Union presented a set of Ethical Framework Principles for Climate Intervention Research including responsible research, inclusive public participation, and transparency (AGU, 2024).

In addition to wide-ranging proposals, certain existing international laws and regulations are understood to provide “hard governance” (i.e., governance mechanisms that are enforceable) for specific aspects of geoengineering. The London Protocol, for example, includes an unratified amendment which mandates that ocean fertilization, a type of CDR, cause no negative effects to the marine environment (Ginzky & Oschlies, 2024). International agreements also offer avenues for “soft governance,” or the adherence to non-binding norms regarding research and deployment around geoengineering. In 2010, for instance, the UN Convention on Biological Diversity passed a non-binding resolution that calls for a moratorium on geoengineering that would affect biodiversity in the absence of “science based, global, transparent and effective control and regulatory mechanisms” (CBD, 2010). Many of these frameworks and principles suggest governance that broadly touches on controlling geoengineering, more deeply studying it, and using it benevolently. That said, they also often lack recommendations for specific enforceable mechanisms.

Academics have also proposed detailed sets of principles aimed at governing geoengineering. The 2010 Asilomar International Conference on Climate Intervention Technologies proposed five principles to promote responsible geoengineering research including noting that it “should be aimed at promoting the collective benefit of humankind and the environment” (Climate Response Fund, 2010). Then in 2013, the Oxford Principles were published and proposed that geoengineering “be regulated as a public good” and advocated for “public participation in geoengineering decision-making” (Rayner et al., 2013). Aiming to address more explicit ethical questions and expand upon the Oxford Principles, the Tollgate Principles were put forth in 2018 (Gardiner & Fragnière, 2018). These principles advocate for democratic participation (principles 1 & 2), appropriate consultation with affected parties (principle 3), and adherence to generally accepted ethical norms like justice and autonomy (principle 9).

Analysing the many proposed principles, Kerryn Brent, Manon Simon & Jan McDonald write that “the priority is to see general principles expounded in instruments that impose binding obligations, such as domestic legislation and institutional research ethics processes, especially in countries where SRM R&D is already afoot” (Brent et al., 2024) While it is relatively easy for general principles proposals to include elements of control, knowledge generation, and benevolent use—it can be difficult for more specific, action oriented governance to do so.

Difficult tasks are still laudable undertakings, and many scholars have proposed specific, action-oriented governance schemes. For instance, Edward Parson and David Keith propose a dual threshold system in which geoengineering interventions above a certain threshold are banned but those below a separate threshold deemed to be of “high-value research” are allowed (Parsons & Keith, 2013). Ralph Bodle et al. offer a similar approach that would prohibit all geoengineering activities with “exceptions under well-defined circumstances” (Bodle et al., 2014). These two proposals most effectively address the issue of controlling geoengineering deployment. They rely in part on knowledge creation and don’t touch upon ensuring benevolent usage.

Another vein of research focuses on whom or what type of organization ought to make decisions about geoengineering deployment. Reynolds notes how many scholars think operational control of geoengineering “should lie with a deliberative intergovernmental institution that counts all or most of the world’s states as members” (Reynolds, 2019). Edward Parson and Lia Ernst, on the other hand, highlight several benefits of a small number of states having the power to control if and when geoengineering is deployed (Parson & Ernst, 2013). Lastly, Simon Nicholson et al. argue in favor of a polycentric control mechanism which would utilize “existing international agreements and institutions” (Nicholson et al., 2018). Again, these proposals focus largely on control and lightly, if it at all, on knowledge creation and benevolent usage.

Daniel Heyen, Joshua Horton, and Juan Moreno-Cruz study an interesting phenomena, though not specifically a governance scheme, which touches both elements of control and benevolence, but not knowledge creation. Their analysis shows that in certain circumstances, counter-geoengineering (i.e., a negation of the free driver problem) can lead to greater inter-state cooperation on deciding whether or not to geoengineer. It can, however, lead to countries using more resources to counteract each other’s geoengineering and counter-geoengineering (Heyen & Lehtomaa, 2021).

Although perhaps less well studied than schemes for control, governance proposals aimed at knowledge creation and dissemination do exist. One interesting proposal calls for “up-stream engagement” with the public, arguing that this kind of early and two-way communication can more closely align the development of geoengineering technology with society’s preferences and desires (Carr et al., 2014). In another vein, Albert Lin argues in favor of an ongoing programmatic technology assessment of all geoengineering research to study the cumulative effects of different proposals (Lin, 2016). Specifically, the assessment would consider these interventions in context of other responses to climate change and their social and systematic impacts in addition to environmental ones. Finally, Jesse Reynolds suggests that governance should leave flexibility for future generations that might have different preferences and knowledge than current ones (Reynolds & Fleurke, 2013).

One well explored aspect of geoengineering governance is the disparity between global North and global South in decision making power and research capacity. Frank Biermann and Ina Möller note how least developed countries are most likely to be negatively impacted by geoengineering but have little to no influence over its research or potential use (Biermann & Möller, 2019). To mitigate this imbalance, they propose four ways to increase least developed nations control over the technology which ideally lead to more benevolent use. Their proposals include increasing least developed countries’ participation in global science networks, using existing UN systems to gain more control, utilizing established multilateral environmental agreements, and finally taking the issue to the international court of justice.

Of the specific, action-oriented governance proposals surveyed here, none thoroughly address all three elements of Mizrahi’s governance requirement. Therefore, understood broadly, the governance literature insufficiently addresses the playing God critique. We believe it is important to respond to the playing God critique for two reasons. First, because “playing God” is a common response from laypersons against geoengineering, designing governance that considers the critique’s core concerns will be important for general societal acceptance and understanding of the technology. Second, governance proposals that address all three components of the playing God critique are stronger than ones that don’t and are thus hopefully more effective and less likely to be rejected.

In the final section, we offer four concrete governance recommendations that are aimed at increasing humanity’s ability to use geoengineering benevolently.

## Geoengineering Governance Aimed at Benevolence

Before introducing our governance proposals it’s important to note that humanity cannot have perfect control, knowledge, or benevolence. These “omni” traits are reserved for the concept of God and impossible to achieve for humans. That said, while we may never achieve perfection, we can achieve better or worse degrees of ability. For example, in general, it’s preferable to seek controlled geoengineering rather than uncontrolled. Designing governance that raises humanity to higher levels of ability – particularly in terms of benevolence – is important because the choices made around geoengineering today will contribute to creating better or worse futures for humanity and the planet.

Additionally, and for fear of stating the obvious, in no future is today’s planetary and socioeconomic status quo maintained. The planetary effects of climate change will increase or decrease, and society will change in accordance. Humankind is already unintentionally and uncontrolledly intervening in the climate, and we may not be able to stop greenhouse gas emissions quickly enough to avoid further and worse climate change.

Therefore, we predict that intentional geoengineering will likely be deployed in the future – either as a temporary “peak shaving” method while fossil fuel use is curtailed and greenhouse gas ultimately removed, or as a permanent solution to ensure the Earth’s temperature remains within livable bounds. Of these two scenarios, the former is preferable since it seeks to solve the underlying problem by removing greenhouse gases as well as stopping, for example, ocean acidification. Conversely, the latter is a band-aid solution which permits continued greenhouse gas emission and places the climate, and the related burden of responsibility, permanently in human hands while not solving problems like ocean acidification.

Drawing from section four, we focus our governance proposals on the third leg of the omni-trait stool: benevolence. We understand benevolence to be the virtue “of being disposed to act for the benefit of others,” and recognize that benevolence is critical for effective governance (Beauchamp, 2019). We aim not to offer a singular, complete governance regime, but rather to expand specific benevolence-related governance proposals that can be incorporated into complete governance mechanisms and to spur a greater focus on benevolence within governance research. While geoengineering proposals often assume benevolence, it absolutely should not be *assumed*, rather, given the fickle nature of human intention, it should be absolutely *explicitly required*. Below we present four governance proposals aimed at ensuring the specifically benevolent research and deployment of solar radiation management geoengineering.

### Specific Benevolence Governance Proposals

#### Explicitly Benevolent

First, we propose that geoengineering research and deployment should be explicitly benevolent. That is, research and deployment activities should carry a statement explaining how they achieve benevolent goals. Control and knowledge have a reciprocal relationship, each empowering the other, as technology and science do, but benevolence must be the foundation of both, and we should be explicit about it. For example, a statement might say something to the effect that “1) Geoengineering is for the sake of common good; 2) its aim is to help people and the environment; 3) it is intended to be temporary; and 4) greenhouse gas reduction should be humanity’s larger goal on the way to climate stability.”

Again, while benevolence is often assumed, requiring that it be made explicit is a simple step that can forestall bad actors and malicious usage. Many sets of principles such as the Calgary Code and the Oxford and Tollgate principles call for the “responsible” use of geoengineering, but mandating that those seeking to geoengineer state how their project serves a benevolent goal is an important step further (Hubert, 2021; Rayner et al., 2013; Gardiner & Fragnière, 2018). Said another way, while SAI technically aims to reduce global surface temperatures by reflecting sunlight, proponents should note that this goal is in service of the larger goal of improving people’s quality of life by reducing extreme weather and heat-related incidents.

One reason that this explicit benevolence is so important is because the idea of using weather or climate as a weapon has already been explored, and therefore contaminates all discussion of the subject (Hamilton, 2013). This malicious use of geoengineering should be overtly and vociferously rejected, perhaps including an international treaty to this effect. Beyond requiring projects to state their benevolent intentions, authorities should also have clear actions to take if a geoengineering intervention deviates from its benevolent goals. One action could be to stop and reverse the geoengineering intervention. Another could be administering compensatory justice.

#### Compensatory Justice

The second governance mechanism we present relates to compensatory justice. Making a concrete causal link between a geoengineering intervention and specific harm is surely difficult, but we believe it could be possible at least in principle and is an important aspect of effective governance. Further, Horton et al. note that geoengineering is compatible with democratic decision making, and therefore democratically deciding to compensate those negatively affected by geoengineering is possible (Horton et al., 2018).

The polluter pays principle (PPP) argues that the agent of geoengineering is responsible for compensating those harmed by it. Toby Svoboda and Peter Irvine argue that the PPP presents overly complex technical and ethical challenges to be useful (Svoboda & Irvine, 2014). Robert Garcia, however, argues that PPP is in fact simpler than Svoboda and Irvine presume and thus is a strong candidate for determining compensation (Garcia, 2014). To make compensation responsibility easier to determine, we propose that those seeking to geoengineer admit that differential benefit and harm will be caused and explicitly commit to providing just compensation when causation can be proven.

Others have argued for “no-fault climate disaster insurance” which would compensate those affected by climate related harms regardless of the mechanism (Wong et al., 2014; Kellogg & Schneider, 1974). One possible avenue would be requiring that any agreement to deploy geoengineering explicitly include a plan and funding mechanism to compensate harmed parties. It’s important to reiterate that no balancing of justice should be based on the balance between the real world and an ideal world, but rather a comparison between the real world and the most likely version of the world that is being avoided by using geoengineering.

#### ELSI Requirement

The third benevolence-centered governance proposal is a requirement of ethical, legal, and social implications (ELSI) research funding. Introduced in 1990 alongside the Human Genome Project (HGP), ELSI research was guaranteed five percent of the total annual HGP budget to study the ethical, legal, and social implications of the project and its related research (Collins, 1999).

The Human Genome Project is looked back on as not only a triumph of science, but a triumph of *good* science, both technically and ethically. ELSI projects helped to set norms regarding human genetic manipulation, reinforce the rejection of human cloning, and established or maintained many other expectations that strongly benefited the social acceptance of HGP discoveries. ELSI research, however, is not uncontroversial. Philip Kitcher argues that the ELSI program focused too heavily on second order social implications and too little on steering the upstream research itself (Kitcher, 2001). Moreso, he claimed that housing ELSI within the HGP disallowed for truly independent critique and analysis.

Building on the National Academies recommendation that geoengineering research funding be tied to accepting codes of conduct, we propose that future geoengineering research funding include a mandate to fund *independent* ELSI research with five percent of the total budget. From a scientific perspective, ELSI research should improve geoengineering implementation because ethical, legal, and social implications are directly tied to its success. Moreover, it would likely make geoengineering more legitimate in the eyes of the public as they would see their concerns being taken seriously.

#### Accelerated Democratic Participation

The fourth governance proposal builds upon previous scholars assertions that geoengineering decisions must be made democratically and with the public’s participation (Jamieson, 1996; Horton et al., 2018; Rayner et al., 2013; National Academies of Sciences, Engineering, and Medicine, 2021). Democratic decision making often yields benevolent outcomes, but we know that it often works slowly. Therefore, to accelerate democracy’s benevolent outcomes we must also accelerate democratic decision making itself – for example by accelerating social conversations around geoengineering. If we anticipate that it might take 5–10 years to have a reasonable social discussion to either approve or deny a geoengineering intervention, it’s important to determine ways to hasten those conversations so they can occur in 2–4 years, or if the situation is especially dire, even 1–2 years. A slow decision making process may lead to no timely decision being made at all.

One model example of accelerating social discussion about geoengineering is the work of The Alliance for Just Deliberation on Solar Geoengineering, a non-profit “fostering just and inclusive deliberation” about geoengineering. Another example from Harry Hilser et al. shows that early and often public engagement on specific geoengineering interventions, especially when considered in addition to local development goals and needs, can improve intervention design and hasten social and legal acceptance (Hilser et al., 2024). In sum, we believe that future governance regimes should emphasize an *accelerated* democratic decision making process.

Given the importance of speed, it is beneficial to accelerate geoengineering conversations both within and outside of governments. Just as hospitals have emergency rooms for urgent care, climate change requires an emergency room for accelerating urgent societal discussions on geoengineering. What might such “democratic climate emergency room” measures look like? First, we can consider why social conversations are slow in the first place. Often there is a lack of expertise on a subject and therefore the public and decision-makers lack the necessary knowledge to inform their decisions. Therefore expertise needs to be funded, including direct research on geoengineering technologies (knowledge), as well as research into control (power and decision-making), and ethics (benevolence).

Developing expertise is expensive, so along with shortening timelines we might consider proportionally accelerating funding. ELSI funding, for example, could be directed towards these accelerated conversations.

## Conclusion

This paper has argued for the relevance of the playing God argument for geoengineering governance. First, we’ve noted that responding to the playing God critique is an important element of geoengineering governance and should be seriously considered. Second, we’ve shown that benevolence is an under-studied governance mechanism compared to control and knowledge. Third, we introduced four such avenues – explicit benevolence, compensatory justice, ELSI, and accelerated democratic participation – which we believe will improve humanity’s ability to use geoengineering benevolently.

As the planet continues to warm, we believe that humanity should consider every tool available to ensure that life remains livable and enjoyable. The best way to achieve this is through dramatic and rapid emissions reduction coupled with a renewable energy transition. If, however, that transition progresses too slowly, geoengineering may be required. In that case, having a robust and effective governance regime is crucial. There are many ways to govern geoengineering, and we believe that an approach that focuses on making the deployment of SAI controllable, based on knowledge, and with beneficent intent is one compelling proposal.

## Data Availability

Data sharing is not applicable to this article as no new data were created or analyzed in this work.
